# Evaluation of donor’s hormonal profile according to sex and age

**DOI:** 10.3389/fphys.2025.1676624

**Published:** 2025-11-19

**Authors:** M. M. de Assis Ramos, F. Y. Ricardo-da-Silva, M. Vidal-dos-Santos, M. J. Vos, P. J. Ottens, G. J. Nieuwenhuijs-Moeke, C. J. Correia, S. J. L. Bakker, L. F. P. Moreira, H. Leuvenink, A. C. Breithaupt-Faloppa, for TransplantLines investigators

**Affiliations:** 1 Laboratorio de Cirurgia Cardiovascular e Fisiopatologia da Circulação (LIM-11), Instituto do Coração (InCor), Faculdade de Medicina da Universidade de São Paulo, São Paulo, Brazil; 2 Department of Surgery, University of Groningen, University Medical Center Groningen, Groningen, Netherlands; 3 Department of Laboratory Medicine, University of Groningen, University Medical Center Groningen, Groningen, Netherlands; 4 Department of Anesthesiology, University of Groningen, University Medical Center Groningen, Groningen, Netherlands; 5 Department of Internal Medicine, University of Groningen, University Medical Center Groningen, Groningen, Netherlands

**Keywords:** hormones, brain death, estradiol, progesterone, cortisol, testosterone, sex, age

## Abstract

Successful organ transplantation depends on several factors, including donor and recipient sex and age. Experimental data show that donor inflammatory status can be influenced by sex hormones, and, after brain death, there are significant differences in organ quality. Sex hormones also influence the immune system during different life stages, for example, during menopause there is a significant reduction in estrogen levels. Thus, the primary aim of this study is to evaluate the steroid profile of human donors after brain death. We performed a retrospective observational case-control study and selected samples from living (LD) and brain-dead (BD) donors from the TransplantLines Biobank and Cohort Study. Donors were stratified by age as Young (Y) from 20–40 years and Old (O), older than 55 years. Serum steroidal hormones from one hundred donors were analysed through LC-MS (Liquid Chromatography-Mass Spectrometry). In BD-females, cortisol and estradiol decreased significantly (*p* = 0.0001) in both age groups when compared to LD. However, an increase in progesterone was seen after BD for older donors (*p* = 0.0001). In BD-males, cortisol decreased significantly in both age (*p* = 0.0001) groups when compared to LD. For testosterone, the results were similar as BD decreased the steroid levels (p = 0.0001) compared to LD in both age groups. In conclusion, our results indicate that steroid hormone levels decrease after brain death.

## Introduction

Clinical evidence suggests that successful organ transplantation depends on several factors, including donor and recipient sex and age (Chambers et al., 2021). Studies have shown that sex-mismatched transplants, particularly female-to-male combinations, are associated with worse outcomes in several organs (Tan et al., 2012; Khush et al., 2012; Coffman et al., 2023). Indeed, a growing body of evidence highlights the role of female sex hormones in the immunological response, a key factor in graft quality and transplant outcomes.

Experimental evidence shows that organ inflammatory status can be influenced by sex hormones (Bonnano Abib et al., 2020) and after brain death induction, females present higher lung (Breithaupt-Faloppa et al., 2016; Ricardo-da-Silva et al., 2021a; Simão et al., 2016; Ricardo-da-Silva et al., 2024; Vidal-Dos-Santos et al., 2025), heart (Ricardo-da-Silva et al., 2020), kidney (Ricardo-da-Silva et al., 2021b) and intestinal (Ferreira et al., 2018) inflammation compared to controls. Additionally, comparing brain dead males and females, a worse inflammatory profile was seen in the females (Breithaupt-Faloppa et al., 2016; Simão et al., 2016; Vidal-Dos-Santos et al., 2025; Correia et al., 2019). These differences could be associated with the acute reduction in female (Breithaupt-Faloppa et al., 2016; Simão et al., 2016) and male sex hormones (Vidal-Dos-Santos et al., 2025) highlighting the impact of hormonal status on the quality of organs from brain-dead donors.

Furthermore, sex hormones also influence immune senescence during different life stages (Khan and Ansar, 2016). Donors have been getting older, and the relevance of age has become more impactful (Dayoub et al., 2018). For instance, menopause results in a significant reduction in estrogen levels (Baker and Benayoun, 2023) and these hormonal changes, along with differences in sex-specific antigens and immune responses, may affect transplant outcomes.

**FIGURE 1:**
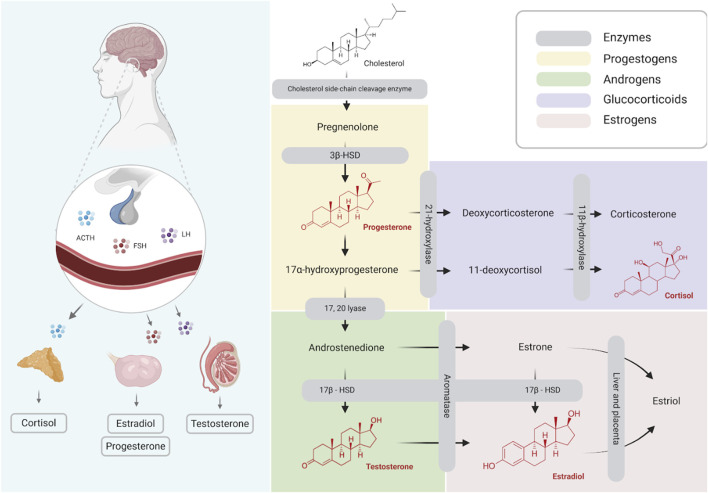
Schematic illustration of steroid hormones synthesis (Created with BioRender.com).

Thus, the primary aim of this study is to evaluate the effects of brain death in the steroid profile of human donors (Figure 1, Steroidal Cascade).

## Methods

### Study design/statement of ethics

This study was designed as a retrospective observational case-control study and used samples from brain-death (BD) transplant donors included in the TransplantLines Biobank and Cohort Study (NCT identifier NCT03272841). It was approved by the Institutional Review Board of the University Medical Center Groningen, Netherlands (UMCG; METc 2014/077), and for which informed consent was obtained from all participants.

### Patient samples

The hormones evaluated were estradiol (E2) and progesterone (P4) in females; testosterone (T) in males and cortisol (Cort) in both groups. The control groups were living donors (LD). The blood samples were obtained from TransplantLines biobank, which collects biological material and medical data from patients before and after organ transplantation, as well as from potential organ donors, both living and deceased. The samples used here were stored at −80 °C (slow freezing trajectory) and aliquots were thawed for the hormone quantifications.

Both sex groups were stratified into young (Y) and old (O). Donors aged 20–40 years were considered Young (Y) and older than 55 years were considered Old (O).

### Analysis

Donor serum was used to measure the steroids via liquid chromatography in combination with tandem mass spectrometry (LC-MS/MS) requiring three different assays. T and P4 were analyzed in 200 µL of serum as previously described (van der Veen et al., 2019). Cort was analyzed in 200 µL of serum as previously described (Werumeus et al., 2016). E2 was analyzed using 200 µL serum as follows: 100 µL [^13^C_3_]-estradiol was added, 500 µL methanol and the samples were mixed for 10 minutes and centrifuged afterwards. The supernatant was extracted using an Oasis MAX µElution Plate 30 µm and eluted with 40 µL methanol. This was evaporated and dissolved again in 110 µL 50% MeOH/H_2_O (v/v %). Fifty microliters were injected on a Waters ACQUITY 2D-UPLC system in combination with a XEVO TQ Absolute mass spectrometer. E2 was analyzed in negative ion mode using 271.1 > 145 as quantifier, and 271.1 > 183 as qualifier. For [^13^C_3_]-estradiol 274.1 > 148 was used as a quantifier and 274.1 > 186 as qualifier. All samples were analyzed in one batch for each analysis.

### Statistics

Statistical analyses were conducted using GraphPad Prism (Version 10.01; GraphPad Software, Inc., La Jolla, United States). Data normality distribution was analysed by Shapiro-Wilk test. Age and BD time were expressed as mean and standard deviation, while hormonal data were expressed as median and interquartile variation. Hormonal data were submitted to rank transformation and analysed by two-way analysis of variance followed by the Benjamini, Krieger, and Yekutieli test for multiple comparisons.

## Results

A total of 100 patients was selected for the study (50 females and 50 males). Among the BD donors, 21 were females and 19 were males. In the control group (Living donors, LD), 29 were females and 31 were males. In females, the most frequent cause of death was bleeding (13 of 21), however, for males, (9 of 19) it was trauma related. Main characteristics are summarized in Table 1.

**TABLE 1:** Demographic variables between donor groups.

Characteristic/ Group	BD				LD			
	Y-F	O-F	Y-M	O-M	Y-F	O-F	Y-M	O-M
N	10	11	10	9	14	15	15	16
Age (years)	31.60 ± 6.45	65.00 ± 7.96	29.80 ± 5.14	69.4 4 ± 7.76	31.93 ± 4.48	61.20 ± 5.05	34.33 ± 3.58	61.25 ± 8.05
BD time (h)	7:33 ± 0:28	7:41 ± 0:25	13:41 ± 9:66	11:49 ± 0:34	—	—	—	—
Cause of death trauma	3	2	9	0	—	—	—	—
Cause of death bleeding	4	9	0	8	—	—	—	—
Other	3	0	1	1	—	—	—	—

Y-F, young–female donors; O-F, old-female donors; Y-M, young-male donors and O-M, old-male donors; BD, brain dead donors; LD, Living donors. Data were expressed as mean and standard deviation.

**FIGURE 2:**
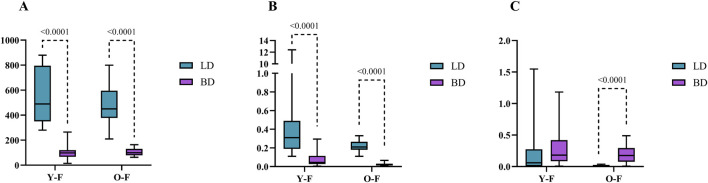
Serum concentrations of cortisol **(A)** estradiol **(B)** and progesterone **(C)** in female donors (nmol/L). Data were expressed as median with min and max. Y-F, Young females; O-F, Old females; BD, brain dead donors and LD, living donors.

In females, cortisol was only altered by BD occurrence (p = 0.0001) with decreased values after BD in both Y [98.25 nmol/L (120.82–66.69)] and O [99.45 nmol/L (130.32–78.99)] groups, when compared to LD in Y [489.4 nmol/L (795.8–350.5)] and O [450.2 (595.1–378.3)] (Figure 2A). Regarding estradiol, values were altered by BD (p = 0.0001) and age (p = 0.0036). In this sense, estradiol decreased significantly under both BD groups, Y [0.045 nmol/L (0.114–0.026) and O [0.018 nmol/L (0.031–0.013)], when compared to LD groups, Y [0.310 nmol/L (0.490–0.190)] and O [0.210 nmol/L (0.265–0.180)] (Figure 2B). Progesterone values were also altered by BD (p = 0.0001) and age (p = 0.0538), but an increase in progesterone was seen after BD for the O group [0.177 nmol/L (0.295–0.076)] in comparison to respective LD [0.012 nmol/L (0.019–0.005)], which was also observed between the Y groups, BD [0.181 nmol/L (0.419–0.085)] and LD [m0.059 nmol/L (0.274–0.005)] (Figure 2C).

**FIGURE 3:**
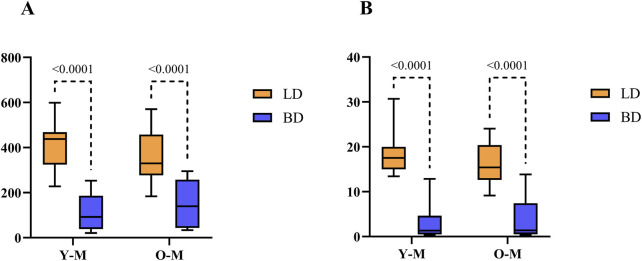
Hormonal quantification of cortisol **(A)** and testosterone **(B)** in males (nmol/L). Data were expressed as median with min and max. Y-M, Young males; O-M, Old males; BD, brain dead donors and LD, living donors.

As shown in Figure 3A, cortisol was also only altered in males by the occurrence of BD (p < 0.0001), whose values were decreased significantly in both Y [92.32 nmol/L (186.48–38.22)] and O [139,98 nmol/L (257.52–43.42)] groups when compared to LD, Y [437.7 nmol/L (468.3–324.1)] and O [329.75 nmol/L (457.95–277.12)]. Figure 3B shows that for testosterone, the results were in line with the steroid levels, as BD values were decreased in Y [1.32 nmol/L (4.64–0.49)] and O [1.37 nmol/L (7.40–0.51)] when compared to LD in both age groups, Y [17.51 nmol/L (19.97–14.98)] and O [15.41 nmol/L (20.38–12.61)] (p = 0.0001).

## Discussion

Our study presents a cohort of BD donors’ hormonal profiles in the Netherlands. Overall, there is a significant decrease in steroid hormones after BD when compared to living donors. Past experimental findings indicate that BD is associated with an acute reduction in hormone levels (Breithaupt-Faloppa et al., 2016; Simão et al., 2016; Vidal-Dos-Santos et al., 2025; Ricardo-da-Silva et al., 2020). As known, hypothalamus and pituitary failure create important hormonal and metabolic imbalances. A longer period of BD may lead to lower levels of steroid hormones due to peripheral consumption of circulating hormones. This pattern is observed for cortisol, estradiol, and testosterone.

We stratified the data by sex and observed that both females and males exhibit this hormonal decline. Regarding age, although older females already present lower hormone levels, a further decrease is still observed. Menopause is a physiological process that occurs between the ages of 45 and 55, during which women experience a decline in mature follicles and estrogen-producing units (Pea et al., 2025). Estrogens exert negative feedback on the production and release of follicle-stimulating hormones (FSH). As a result, perimenopausal women often present elevated FSH levels, which may lead to an abnormally high maturity rate of developing follicles. In contrast, luteinizing hormone (LH) levels generally remain within the normal range (Larsson-Cohn, 1985).

One important variable to be considered within the groups is the donor’s cause of death. The different aetiologies of BD, whether slow or fast, lead to varying degrees of organ-specific injury (Vidal-Dos-Santos et al., 2025; Rebolledo et al., 2016; van Zanden et al., 2020). This factor must be considered when evaluating steroid hormone behaviour because regional blood flow in the hypothalamus during BD suggests that there may be minimal and slow flow sufficient to maintain the integrity of part of the hypothalamus, enabling at least some hormone transport (Arita et al., 1993). For instance, Arita et al. (1993) observed severe hypocortisolism in only 23% of BD cases. Although in our study the cause of death did not appear to influence hormonal status, this remains a key factor to consider.

The effects of steroids are modulated by stress, which provides energy through cholesterol and other lipid derivatives, promoting 17β-hydroxysteroid dehydrogenase activity. In hemorrhagic shock, increased secretion of adrenocorticotropic hormone (ACTH) may stimulate cortisol production. It is known that adrenal androgens can be aromatized into estrogens (Christeff et al., 1988). Unlike brain-dead patients, male patients in shock also show elevated estradiol levels and reduced testosterone levels (Christeff et al., 1988). Furthermore, decreased serum testosterone in critically ill males and postmenopausal women have been linked to reduced 17β-hydroxysteroid dehydrogenase activity and/or increased aromatization (Spratt et al., 1993). These factors contribute to the reduced testosterone levels observed in males after BD.

It is relevant to consider the half-life of the primary analysed steroids. Progesterone has a half-life of approximately 5 minutes in the body (Taraborrelli, 2015), testosterone remains in circulation for 60–80 min (Hellman and Rosenfeld, 1974), and cortisol has a reported half-life of 76.5 min (Hindmarsh and Charmandari, 2015). Estradiol, when administered orally at a dose of 4 mg, has a mean half-life of 13.5 ± 4.4 h (Kuhnz et al., 1993). Hormonal differences may result from metabolism: progesterone is rapidly reduced into other steroid precursors, while estradiol is synthesized later from testosterone and estrone (Figure1). It is possible that during stress of the BD onset, the adrenal glands synthesize great amounts of allopregnanolone (Purdy et al., 1991), which would be converted to progesterone.

Our initial data support previous observations from experimental studies; however, studies with larger populations are needed to validate these findings. Further studies assessing different age groups, particularly among women, are necessary to determine the variation in hormone levels throughout life. Unfortunately, interfering variables such as hormone therapy and medication intake, which could influence final donor serum concentrations, are not available for evaluation in this study. One important variable to be considered within the groups is the donor’s cause of death.

In conclusion, our results suggest that steroid hormone levels decrease after BD. Future studies evaluating the hormonal profiles of donors may provide greater insight into donor management and organ quality.

## Data Availability

The raw data supporting the conclusions of this article will be made available by the authors, without undue reservation.
